# Having conversations about alcohol in frontline healthcare services: Experiences and perceptions of consumers and healthcare staff

**DOI:** 10.1371/journal.pgph.0006893

**Published:** 2026-08-19

**Authors:** Brooke Hayward, Sarah Herbert, Sarah Sharpe, Luisa Silailai, Hinewai Pomare, Erin Eydt, Kimberley Tangaere

**Affiliations:** 1 Research and Evaluation Office, Ko Awatea, Counties Manukau Health, Auckland, Aotearoa New Zealand; 2 Pacific Inc trading as Le Va, Auckland, Aotearoa New Zealand; 3 Te Kupenga Hauora Māori, Faculty of Medical and Health Sciences, University of Auckland, Auckland, Aotearoa New Zealand; 4 Population Health, Counties Manukau Health, Auckland, Aotearoa New Zealand; PLOS: Public Library of Science, UNITED STATES OF AMERICA

## Abstract

Alcohol-related harm is a global public health priority. To address a gap in the provision of screening, brief intervention and referral for treatment (SBIRT) in front-line healthcare services, a district health organisation based in South Auckland, Aotearoa New Zealand, adapted and implemented a model of SBIRT called the Alcohol ABC Approach within general practices, maternity services, hospital emergency care, an inpatient ward, an outpatient clinic, and the district-wide community smoking cessation service. The Alcohol ABC approach aims to identify hazardous or harmful alcohol use in adult populations through routine, non-targeted assessment, provide brief advice and support to reduce alcohol consumption, refer people to support services where indicated, and prevent alcohol-related harm. A non-experimental, mixed methods, process and outcome evaluation examined the implementation, quality and outcomes of the programme to inform future delivery. Reflexive thematic analysis of semi-structured interviews with consumers (n = 8) and staff (n = 19) explored experiences and perceptions of conversations about alcohol and implementing the Alcohol ABC Approach. Findings highlight how social norms surrounding alcohol shape the acceptability of conversations, while relational practices grounded in whanaungatanga (relationship-building) and manaakitanga (care and respect) enable more meaningful dignity-enhancing engagement. Participants described conversations about alcohol as increasing awareness, supporting behaviour change, and challenging the normalisation of hazardous drinking. However, implicit and explicit biases, particularly ethnic bias, influenced who was asked about alcohol and how these conversations unfolded. Organisational support was essential for building staff skills and confidence to initiate discussions. These findings highlight the importance of equity-focused, culturally safe SBIRT approaches that address power and bias, and centre relationships in clinical encounters. While grounded in the context of Aotearoa New Zealand, this study offers insights for adapting and implementing alcohol harm reduction interventions in diverse settings, particularly those seeking to integrate decolonising and anti-racist principles.

## Introduction

Alcohol-related harm is a global public health priority [1]. In Aotearoa New Zealand (henceforth Aotearoa) there are high rates of hazardous alcohol use [2] as measured by the Alcohol Use Disorders Identification Test (AUDIT). This test measures frequency and quantity of alcohol consumption using a scoring method. A score of eight or more represents hazardous drinking, i.e., “an established pattern of drinking that carries a high risk of future damage to physical or mental health” [3, Hazardous Drinking Section]. One in six adults (16.0% in 2022/23) in Aotearoa engages in hazardous drinking [3]. Mirroring global trends, the burden of alcohol harm is experienced inequitably among males, young people (18–24 years), and people living in the most socioeconomically deprived areas [3–5].

Māori (an indigenous person of Aotearoa) and Pacific people experience disproportionate alcohol-related harm due to the unfair and unjust distribution of social, cultural, political and economic determinants of health, including the enduring effects of colonisation, racism and socio-economic deprivation [6–8]. Crucially for Māori, persistent inequities are associated with the ongoing impacts of colonisation which have contributed to significant land loss, cultural marginalisation, racism, discrimination, and socio-economic deprivation, and represent failures by the Crown to honour te Tiriti o Waitangi (a significant agreement between the British Crown and Māori chiefs made in 1840) [8]. This important historical context is further elaborated in S1 Text, and a glossary of te reo Māori (the Māori language) in S2 Text. Within these structurally constrained contexts, patterns of hazardous alcohol use are more likely to emerge among those who consume alcohol. Hazardous drinking is higher among Māori (25.1% in 2022/23) compared to other ethnic groups in Aotearoa [3]. While Pacific peoples are less likely to drink alcohol than non-Pacific peoples in Aotearoa, those who do consume alcohol are more likely to engage in hazardous alcohol use, and experience disproportionate harm [3,9]. Similarly, alcohol-related harms are disproportionately higher for Māori compared to non-Māori [10].

The World Health Organization’s ‘SAFER’ recommendations for the prevention of alcohol-related harm include the most cost-effective strategies or ‘best buys’, which are: strengthening restrictions on alcohol availability in communities, adopting comprehensive restrictions on alcohol advertising and sponsorship, and raising prices of alcohol through excise taxes and pricing policies [11–15]. Alongside these impactful preventative strategies, it is also important that people have equitable access to high quality and culturally safe healthcare services to identify and support the prevention of harmful alcohol use. Screening, brief intervention and referral for treatment (SBIRT), also part of the ‘SAFER’ package [11], is an individual-level, evidence-based best practice intervention for reducing alcohol harm [11,16–18]. It aims to identify hazardous or harmful alcohol use; provide feedback, information and advice on reducing alcohol consumption; provide support to those who need it; and prevent alcohol-related harms [16,17]. Implementation within an equity framework is crucial [4,5,12].

To address a gap in the provision of SBIRT in local front-line healthcare services, Counties Manukau Health (a district health organisation serving, at the time of this study in 2020, a population of approximately 580,000 people in South Auckland and surrounding areas) developed a programme of work to adapt and implement a model of SBIRT called the Alcohol ABC Approach. This approach involves **A**sking consumers about their alcohol use and **A**ssessing use with the Alcohol Use Disorders Identification Tool for Consumption (AUDIT-C) [19]; providing tailored **B**rief advice; and referring for **C**ounselling to alcohol support services if indicated [20]. Adapted from smoking cessation practice in Aotearoa, the purpose of the Alcohol ABC Approach is to support healthcare staff to have skilled and empathetic conversations with people and families about alcohol. It provides a systematic way of embedding alcohol assessment and advice across the health system and within everyday practice of front-line healthcare professionals who are not specialist mental health and addiction practitioners.

Between 2016 and 2020, the Alcohol ABC Approach was implemented within a range of settings in Counties Manukau (CM) including general practices, maternity services, hospital emergency care, an inpatient ward, an outpatient clinic, and the district-wide community smoking cessation service. Little is known, both in Aotearoa and globally, about the application of the Alcohol ABC Approach in these settings.

Programme staff (those in dedicated programme roles) worked collaboratively with healthcare services staff using project management and quality improvement methods to adapt the model to each setting, develop enabling systems and processes, and provide customised training and sustained support. Common components across projects included: identifying champions in each setting, adapting existing processes and forms (including electronic enablement) to embed alcohol assessment, provision of information and collateral regarding specialist services for referral of patients/clients, training for staff on ‘having conversations about alcohol’, and monitoring and evaluation.

A non-experimental, mixed methods, process and outcome evaluation was undertaken to describe and understand outcomes and quality aspects of the programme over the first four years (2016–2020), to strengthen future work. This paper describes the qualitative process component of the evaluation, which explored consumer and staff experiences and perspectives of having conversations about alcohol and implementation and delivery of the Alcohol ABC Approach.

## Methods

This qualitative process evaluation used semi-structured interviews with consumers and staff to explore their experiences and perspectives of having conversations about alcohol, and delivery of the Alcohol ABC Approach. A conscious decision was made to prioritise partnerships with Māori and to ensure a focus on the implications for Māori regarding conversations about alcohol. This position recognises both: (i) the rights-based imperative for Counties Manukau Health, as a Crown agency, to uphold their commitments to te Tiriti o Waitangi - requiring a sustained reduction in alcohol harms experienced by Māori, to achieve equity of outcomes consistent with non-Māori in Aotearoa, and also; (ii) a needs-based approach to address inequity. Multiple actions were taken to embed this position across the evaluation methodology (e.g., recruitment, interview approach), including privileging the relevance to Māori within evaluation findings.

### Data collection

Semi-structured interviews were undertaken with two key participant groups: (i) people who had been assessed for alcohol harm (consumer participants), and (ii) healthcare staff delivering alcohol harm assessments and having conversations about alcohol with consumers (staff participants). All interviews were conducted throughout August and October 2020.

Interview schedules included a series of open-ended questions, and closed response questions to collect participant demographics. Interviews with consumer participants explored: (i) perceptions and experiences of being asked about alcohol, (ii) perceptions about the value and importance of being asked about their alcohol use, (iii) whether they received the support/help they needed (where relevant) or experiences of changes to alcohol use or alcohol-related harm as a result of conversations, (iv) observations of alcohol-related harm in their life or community, and (iv) what could be improved about the Alcohol ABC Approach delivery by healthcare staff. Face-to-face interviews were feasible with staff participants under COVID-19 restrictions. Interviews undertaken with staff were guided by a semi-structured interview protocol incorporating the following topics: (i) perceptions and experiences of implementing the Alcohol ABC Approach (i.e., what worked and what didn’t, and what could be improved); and (ii) perceptions and experiences of having conversations about alcohol with consumers.

Interviews were grounded in tikanga (Māori customs); welcoming participation from whānau (extended family), prioritising whakawhanaungatanga (the process of building culturally meaningful connections), providing koha (gift or token of appreciation), and committed to open and honest kōrero (conversation) in which the validity of people’s experiences were accepted free of judgement. Two programme staff members were provided with qualitative interview training facilitated by evaluators. Participants who identified as Māori were interviewed by a Māori programme staff member who was able to facilitate kōrero in te reo Māori (if they desired). All other consumer participants were interviewed by a Pacific programme staff member. ‘Pacific’ is a grouped ethnicity classification used in this context for de-identification. It incorporates many Pacific ethnicities, including for example, Samoan, Niuean, Fijian, Tongan, Cook Islands and more. We recognise that Pacific peoples are a diverse group with unique ethnic and cultural identities. Face-to-face interviews were planned to ensure whānau inclusion. However, due to COVID-19 restrictions at the time, interviews were required to be conducted via telephone.

Consumers were eligible to participate if they were at least 16 years of age, usually resided in CM, had experienced having a conversation about alcohol with their care provider in the twelve months prior to evaluation participant recruitment, and could recollect the experience. Staff were eligible if they had experience delivering the Alcohol ABC Approach and having conversations about alcohol with consumers during the same period. Maximum variation sampling was used to select diverse consumer participants across a range of settings and demographics (i.e., service used, age, gender, ethnicity). We intended to recruit 20 consumers (at least 50% Māori). An expert sample of staff participants aimed to include staff involved in Alcohol ABC Approach implementation and delivery. Interviews were audio recorded and transcribed for analysis.

### Participants

Eight consumers and nineteen staff participated in qualitative interviews. Consumer participants were recruited from the following healthcare settings: general practice (GP) (n = 3), emergency department (ED) (n = 2), community smoking cessation service (n = 2) and outpatient clinic (n = 1). There was a lower-than-intended proportion of Māori (n = 3, 40%) (further detailed in limitations), and interviewers observed an over-representation of participants with higher AUDIT-C scores.

Staff participated from the following settings: GP (n = 8), ED (n = 4), outpatient clinic (N = 2), an in-patient ward (n = 1), maternity services (n = 2), and smoking cessation service (n = 2). The eight GP participants included three clinicians and five primary health organisation (PHO) staff who were project champions. PHOs are community-based organisations that provide primary health services in Aotearoa, either directly or through contracted providers [21].

### Data analysis

Qualitative data transcripts were uploaded to NVivo for analysis. Reflexive Thematic Analysis (RTA), following Braun and Clarke [22], was used to generate themes across data. This approach emphasises the active role of the researcher in knowledge production, recognising that themes are constructed through engagement with the data, rather than discovered. The analysis approach was guided by health equity frameworks, aligned with the strategic priorities of the healthcare organisation. These frameworks provided a critical lens for examining structural and relational influences on alcohol conversations, and informed interpretation, reflexive discussion, and analytic decisions throughout.

Transcripts were initially coded by two authors. SH (a Māori health researcher) served as the primary coder for transcripts contributed by consumer participants who identified as Māori, while BH coded transcripts from non-Māori participants. Themes were then developed by BH. Reflexive interpretation was supported through explicit acknowledgement of researcher positionality (cis-gendered, heterosexual Pākehā - New Zealander of European descent -, tāngata Tiriti - non-Māori person of the Treaty -, and mother) and ongoing discussion with co-authors and Māori health equity specialists (SH and KT) to further refine themes. Coding and theme generation involved an iterative, sequential process, with themes being refined, expanded or collapsed through collaborative debriefs. Analytical decisions were grounded in Te Ao Māori (Māori worldviews or perspectives) and health equity principles, enhancing the cultural validity of findings for Māori. Inconsistencies in interpretation arising from different cultural worldviews among authors were identified and explored. Where such inconsistencies were detected, Māori co-authors had final interpretive input.

Data from consumer and staff participants were initially analysed separately to preserve group-specific perspectives. Consumer participant data were prioritised in the final analysis to amplify the voices of those receiving care. This was considered important in the context of a relative gap within the literature exploring consumer perspectives of SBIRT specifically. Together with Māori health equity specialists (SH and KT), staff data were subsequently integrated, which reinforced consumer themes. For example, consumer perceptions of discomfort around discussing alcohol use were reflected in staff reports of similar hesitancy. While nuances specific to each group were maintained, and references to the source of perspectives (consumer, staff or both) are reported where relevant, this combined approach supports a richer understanding of barriers and facilitators to conversations about alcohol while acknowledging the power dynamics inherent in a healthcare environment influenced by medical hegemony.

### Ethics statement

This evaluation complied with National Ethics Association Committee (NEAC) Standards for health and disability research and evaluation in Aotearoa, and Aotearoa New Zealand Evaluation Association standards. The National Health and Disability Ethics Committee (HDEC) deemed this programme evaluation out of scope for review.

All participants provided verbal (phone interviews) or written (face-to-face interviews) free and informed consent prior to participation. This process was facilitated by programme staff (LS and HP) who first contacted potential consumer participants to describe the evaluation and its purpose (note that programme staff were not clinicians responsible for undertaking conversations about alcohol with consumers). Potential staff participants were identified from programmatic records and roles, and were recruited by BH, who extended an invitation to participate via email. Staff and consumer participants were provided with both a verbal explanation of the study and written information outlining the aims of the evaluation, why they had been approached, how their information would be used, and key data management practices in accordance with the Privacy Code in Aotearoa. All potential participants were given the opportunity to ask questions and consult with others if desired. As consumer participants were reached via telephone, they were able to request a later telephone follow-up, decline to participate in the evaluation, participate at the time of call, or schedule a preferred interview time. Verbal consent for participants participating via phone was captured on audio record at phone interview commencement, reflecting adaptations necessitated by COVID-19 restrictions. Staff consent was documented in written form at interview commencement. All evaluation participants were aged 16 years or older, consistent with HDEC guidance that individuals in this age group can provide informed consent without parental approval.

## Results

The results are presented as a narrative description of key themes related to experiences of having conversations about alcohol. Five key themes are presented:

- Key theme one – Alcohol use “is a normal thing”: Social norms shape behaviour change, and conversations about alcohol.
- Key theme two – Alcohol use “is not a subject any stranger asks”: Whanaungatanga (relationships) and manaakitanga (hospitality) facilitate mana-enhancing conversations. Mana is a fundamental concept in Māori culture, representing authority, power, and spiritual strength. The concept of mana has complex and multiple meanings and in the context of this document ‘mana-enhancing’ refers to actions, practices, and policies that aim to uplift, empower, and respect the dignity, authority, and identity of individuals or groups, particularly within the context of Māori culture in Aotearoa [23].
- Key theme three – “It’s not just youth that are binge drinking”: Recognising implicit bias in conversations about alcohol.
- Key theme four – “I need my health”: Conversations about alcohol are valuable and important.
- Key theme five – “We are learning it is okay to ask”: Staff confidence to approach conversations about alcohol.

### Key theme one - “It’s a normal thing”: Social norms shape behaviour change and conversations about alcohol

Consumer and staff narratives reveal that alcohol use is widely perceived as socially normal, shaping how risk, responsibility, and readiness to change alcohol use are understood. Individuals often minimise the severity of their own or others’ alcohol use, framing it as socially acceptable or integral to social life. Readiness to change alcohol use behaviours is shaped within complex relational, social, and environmental contexts. This theme highlights a tension between dominant narratives of individual responsibility and the broader societal and structural factors influencing alcohol use and behaviour change.

Consumer participant accounts highlight how alcohol use, including heavy or regular consumption, is socially normalised, shaping how individuals interpret risk, responsibility and the perceived need for change:

“I have been around drinking all my life… I’d say it probably has been just a normal [part of my life]. It’s just not a big thing in my life, it’s a normal thing” (PP04).

“I think it is pretty normal for lots of people to get really [drunk]. I just drink to get wasted. I think that’s a big culture in Auckland, maybe in New Zealand” (PP05).

Consumer participants also expressed an awareness of, and anxiety about, alcohol use that they believe others might perceive as unacceptable. Their narratives link problem alcohol use to a person’s functionality and attempt to distance themselves from socially unacceptable alcohol use, with the ability to perform everyday activities serving as a marker of socially acceptable alcohol use.

“I know that I probably drink more than the average person, I know that for sure … I think there’s a lot of people out there [who are] functioning alcoholics… I think people think I’m an alcoholic, but I think of [alcoholics as] some damn, inept bloody, you know, person that’s just derelict, you know, wakes up in the morning and drinks” (PP04).

Consumer participant narratives highlight a disconnect between medical and lay interpretations of ‘hazardous’ alcohol use. Moreover, they suggest that societal normalisation of some alcohol use, such as heavy and/or binge drinking, may diminish individuals’ perceived urgency or need to change their alcohol use.

“I probably do overindulge. Then again, it could be worse. I mean, I do like to have a glass of wine or a couple of beers or whatever … But I do think having alcohol is sort of like, you’ve got to have alcohol, you’ve got to have it there” (PP01).

“I personally haven’t really been harmed by anyone who is [drinking]… I don’t feel like I’m in danger, or a lot of danger from people who are drinking a lot, but I do kind of think that it’s a big binge drinking culture” (PP05).

In the above quotes, consumer participants minimise the severity of their alcohol use by emphasising “it could be worse” and framing alcohol as an acceptable and integral cultural artefact.

Staff narratives mirrored these patterns, often minimising alcohol use and framing it as normal. This is illustrated in the following quote, which captures staff surprise at the level of alcohol consumption that may be considered hazardous:

“[It is] quite a low level [of alcohol consumption] before you get above the guidelines… My colleague at work, he was quite surprised that he was above the guidelines and that sort of thinking. But I don’t think they have worrying drinking … ” (S11).

Similarly to consumer participants, staff participants also sought to position their own drinking as normal, minimising a rationale for change: “I don’t think [staff] have worrying drinking”*.* The normalisation of harmful alcohol use impacted on participants’ feelings of personal ‘readiness’ to change, and ‘readiness’ for treatment of alcohol-use disorders (where applicable).

Consumer participants who self-reported riskier alcohol use described proactively initiating conversations about their alcohol use by offering information to healthcare staff, directing conversation to this subject, or independently seeking support services. They described taking an active role in initiating conversations with healthcare staff, rather than being identified through routine screening, which they framed as a turning point in seeking support. “It was me. I steered it all, I just had enough. I’d had enough [of drinking]” (PP02). This account reflects the participant’s readiness to engage in conversations and seek support, and demonstrates agency in navigating healthcare interactions.

However, such expressions of agency were embedded within broader narratives of cumulative stress, relational strain, and environmental constraint, rather than framed as a simple or autonomous decision to change alcohol use. As one participant noted:

“I don’t think you can just decide ‘I want to give up drinking’… I know it’s not that simple” (PP04).

This reflection situates readiness within relational and contextual realities, including family, social obligations, and environmental pressures, highlighting that change is sustained in response to lived circumstances rather than solely from individual will.

Across consumer and staff participants, readiness to change alcohol use was embedded within social, relational, and environmental realities. Consumer participants with previous alcohol support experiences often attributed their failure to sustain reduced alcohol use to a lack of personal readiness, while also recognising a range of social and environmental influences shaping their alcohol use, such as easy access to alcohol, work and financial stress, and social norms:

“I’d been to [my doctor] before and told her that I had a bit of a problem with the drinking and she sent me off to [an alcohol support service] so I went there and I did programme there. And then in all honesty, I wasn’t really ready to properly give it up” (PP02).

In elaborating on this experience, the above participant goes on to describe their alcohol use as a primary coping mechanism within the context of significant financial and personal stress at home, work pressure and stress, COVID-19 disruption, shame, loneliness, and parental guilt. Further, they described ongoing participation in social environments where alcohol was central to celebrating, alongside ease of access to alcohol in their community. Despite articulating these broader contextual influences, participant narratives frequently returned to self-pressure to “be able to do it for myself” and self-blame for failing: “I just need to try a bit harder” (PP02).

This pattern of internalising responsibility for change, even while recognising substantial social and environmental constraints, highlights a tension within consumer participant accounts between lived, contextual realities and dominant narratives that position readiness and sustained behaviour change around alcohol use as matters of individual responsibility.

These dominant narratives were also embedded in service delivery, with staff framing support as contingent on patients “wanting to do it” (S16) themselves rather than being actively facilitated:

“It is up to them to seek further support and to drive the process further rather than us spoon feeding it to them” (S07).

### Key theme two - “It’s not a subject any stranger asks”: Whanaungatanga (relationships) and manaakitanga (hospitality) facilitate mana-enhancing conversations

Consumer and staff narratives highlight that the quality of relationships strongly shapes conversations about alcohol use in healthcare settings. Practices demonstrating whanaungatanga (relationships) and manaakitanga (hospitality and care) foster safe, non-judgemental, and culturally affirming interactions. Positive relational contexts, including ongoing trust and familiarity, enable consumers to disclose alcohol use and engage in discussions, while short or transactional encounters require deliberate strategies to build connection. This theme highlights that relational and culturally grounded practices are central to mana-enhancing healthcare experiences and effective Alcohol ABC approaches.

Consumer participants highlighted the risk of feeling judged or persecuted by healthcare staff, and/or society more broadly, about their alcohol use:

“I sort of felt edgy answering [the Audit C questions]… You start to get to think like people might be judging you because of your answer” (PP03).

‘How’ they were asked was important for consumer participants. Staff practices that demonstrate whanaungatanga and manaakitanga were described as potentially protective for participants, facilitating safer, non-judgemental conversations about their alcohol use. In the context of healthcare, mana- enhancing practices might involve culturally responsive care that respects and incorporates Māori values and world views, ensuring that healthcare services are not just accessible and clinically safe but also culturally affirming and empowering. This approach contributes to better health outcomes by fostering environments where individuals feel respected, valued, and supported.

For manaakitanga, consumer participants reported effective staff practices to include: being warm and friendly, ensuring privacy and confidentiality, taking a casual approach without ‘forcing’ the conversation, acknowledging awkwardness, understanding health priorities, identifying underlying causes, following up, and providing appropriate support.

The relational context of being asked was also emphasised, “it’s not a subject that any stranger asks you...” (PP03). When alcohol conversations occurred within a positive existing relationship, for example one in which whanaungatanga has occurred, this supported mana-enhancing experiences:

“I have a really good relationship with my doctor. I have been with her for years, say 20 years I think... So I felt a bit embarrassed telling her, but she was so understanding and so gentle and not judging me.” (PP02).

The value of a relationship to facilitate conversations and action on alcohol was echoed by staff:

“I had a young mother that I was looking after, and she had disclosed to me, not the first time that I had met her, but when I developed a relationship with her, she disclosed that she was drinking in her pregnancy and was finding it hard to cut down on her drinking. I was able to support her on that pathway…” (S04).

Staff participants reported practicing whakawhanaungatanga (the process of building culturally meaningful connections) with consumers prior to approaching conversations about alcohol use in the following ways: maintaining a conversational approach, connecting through shared experiences, using humour to put them at ease, valuing whānau voice and inclusion, and prioritising face-to-face conversations. However, these practices missed opportunities to draw on their understanding of Te Ao Māori, with a lack of self-disclosure about their own whenua (land) connections and whānau. Further, consumer participant narratives offer limited examples of whānau being included in conversations about their care, and/or alcohol and support pathway steps.

Staff experiences demonstrate that certain health settings characterised by longer consumer stays or repeated visits (e.g., GP, smoking cessation service, maternity service or inpatient wards) enabled whakawhanaungatanga and whanaungatanga to occur. This provided flexibility in staff decisions to engage, pause, delay and return to conversations about alcohol use at different times. Conversely, short stay settings (e.g., hospital emergency care) required staff to adopt alternative strategies, including initially dedicating time to connect, clarifying consumer needs or concerns, and returning to alcohol conversations later within the same interaction.

### Key theme three - “It’s not just youth that are binge drinking”: Recognising implicit bias in conversations about alcohol

Staff narratives reveal that implicit biases related to age, sex, and ethnicity shape how alcohol use conversations are conducted. These biases influence how, and with whom staff engage, potentially limiting equitable care and reinforcing stereotypes. Time constraints, self-consciousness, and uncertainty about culturally responsive practice further compound these inequities. This theme highlights the importance of recognising and addressing implicit bias to ensure that alcohol conversations are respectful, culturally safe, and equitable across all consumer groups.

Biases related to individuals or groups based on age, sex and ethnicity are embedded within staff narratives about their experience approaching conversations about alcohol. For example, younger people as a group were identified by staff as having hazardous behaviours around frequency of drinking, which for the following participant, formed a generalised and myopic interpretation of young people’s cognitive capacity: “When you’re younger you drink often and you don’t really think about it” (S16).

Ethnic biases held by staff are highlighted in beliefs that drinking is the inherent nature of some groups (“it is in their system”), the difficulty staff may experience in breaking these patterns of thinking (“it’s really hard at first, you always think…”), or in the way they may feel shocked, surprised, or curious about consumers they interact with who challenge or contradict their held biases:

“… Unexpectedly, quite a few of them [people who identify as - ethnicity removed -] are quite big drinkers, especially the women interestingly enough, so we use an interpreter… It’s surprising, yeah it is surprising the people that you least expect to drink, [actually drink] a fair bit” (S08).

“It’s really hard at first, you always think, ‘they will just drink again because it is in their system’, but in my mindset, as long as I give the support that even if they drink, just give this number a call …” (S19).

Staff insights demonstrate how implicit biases can cause negative impacts on consumer care; shaping ‘if’ and ‘how’ staff engage with consumers in conversations about alcohol. For example, withholding of care occurred when staff inadvertently centralised themselves, prioritising their own comfort, insecurities (e.g., “feeling self-conscious about asking” (SO7) or “it can be a little bit intimidating” (S14)), time and clinical demands, above consumers’ care rights and needs. Time constrained environments led some staff to not ask consumers from some ethnic groups about their alcohol use, because they perceived that these groups would have hazardous alcohol use requiring brief intervention, and were concerned they wouldn’t have time in their consultation to offer brief advice.

Many staff found it challenging to articulate how they took someone’s culture into consideration when approaching conversations about their alcohol use. Some staff perceived that attending to culture was natural and struggled to explicitly explain how they do this, some needed time to think and reflect on their practice, while others weren’t sure if they had answered the question correctly:

“Um, well, I, I’m, I’m, I’m not sure, um, I guess, having grown up in New Zealand and I know the ways, I know about lifestyles… It sort of comes somewhat naturally now” (S03).

Tailoring approaches based on cultural or ethnic identity was not something all staff were confident with or considered a priority. Moreover, staff narratives conflate and use the terms ‘culture’ and ‘ethnicity’ interchangeably:

“…[I] see the person as a whole person and not their race or sex or whatever they may be and not specialise in other areas because they’re actually causing discrimination amongst other groups…I’d like to see it blended right across all cultures, not between one or two singled out to be equitable” (S02).

### Key theme four - “I need my health”: Conversations about alcohol are valuable and important

Consumer and staff narratives emphasise that conversations about alcohol are valuable and important for health and wellbeing. Consumers recognised these discussions as a catalyst for reducing alcohol-related harms and improving broader health outcomes, while staff highlighted the potential for alcohol conversations to support holistic care and public health objectives. The perceived value of these interactions reflects both the direct impact on individual behaviours and the wider influence on whānau, and community outcomes. This theme highlights the value of routine and culturally safe alcohol conversations as a component of effective public health practice.

All consumer participants considered having conversations about alcohol is key. They particularly recognised value in these conversations because of how their alcohol use impacts on other behaviours (e.g., smoking) and overall health and wellbeing:

“[I want this] especially for health. It’s not about money. It’s not about everything, I just want help, I need my health. I want to go back like before” (PP04).

The perceived importance of conversations about alcohol were also linked to participants’ observations of harms caused by alcohol use, either directly, or observed in their community. Examples raised included drinking and driving, exacerbation of relationship stress, financial stress, family harm (including physical violence), intergenerational harm, and other substance abuse.

Consumer participants who were observed to have lower AUDIT-C scores did not report changes to their alcohol use as a result of being asked about their alcohol use, while those who identified as having hazardous alcohol use reported conversations to be a catalyst for change. Self-reported changes included reduced frequency (e.g., taking a night off from drinking), reduced volume (e.g., two beers instead of a box) and changing alcohol type (e.g., wine and beer instead of spirits). These changes were also reported to reduce alcohol-related harm, including whānau relationship stress, or financial stress.

Similarly, staff participants consider the Alcohol ABC Approach important, alongside public health measures.

“It’s just integral. [If] you’re changing one health behaviour … we may as well hit all health behaviours as we’re at it… Alcohol has such devastating effects and is often underrated, and we’ve got this big opportunity to go in” (S10).

Staff perceived value in the potential for the Alcohol ABC Approach to support a more holistic health assessment that addresses the underlying and intersecting causes of health service presentation, and deliver follow-up support. The ability for diverse reach within the community through their services also made staff participants from ED settings feel the approach was critical:

“I probably was in the mindset of I know they are drinking, that’s not an us thing, that’s, the ward will ask them… Then thinking about it, people that come to the front door, about 80 percent of them only ever get to the emergency department. So I probably realise that we probably do have more of a vital role” (S09).

### Key theme five: “We are learning it is okay to ask”: Staff confidence to approach conversations about alcohol

Staff narratives highlight that building confidence and skills is central to initiating conversations about alcohol use. Many staff initially felt nervous, hesitant, or concerned about consumer reactions, but over time developed confidence through training, mentoring, and repeated practice. This theme illustrates the transformative journey from uncertainty to competence, demonstrating that staff capability-building is a key enabler for the effective delivery of the Alcohol ABC Approach.

Staff participant narratives indicate that they initially felt nervous, reluctant, or awkward about initiating conversations with consumers regarding alcohol use:

“When you first start doing something you don’t always feel that confident in asking questions and it can be nerve-wrecking having to ask people personal questions about alcohol use and things. For me, doing it more often and becoming a champion and speaking to other nurses in handovers about it and explaining to them how to do it, and also helping people on the floor when I’ve been coordinating an area and prompting other nurses and giving them a hand in doing it, explaining to them what advice they can give – that’s helped me do it better as well. Being able to teach other people how to do it so they can go on to gain confidence as well is the main thing” (S09).

“I think it was more learning how to have these conversations and not be scared about people’s responses to it. Initially when it was in the piloting stage, and when it first became business as usual, there was a lot of hesitation about, ‘what if they’re going to get defensive about it’. It was reassuring ourselves that it was okay, that even if they were defensive about it, that we still could fall back on addressing it in a future session” (S17).

These quotes highlight the transformative journey many staff have experienced, from initial uncertainty and hesitation about discussing alcohol, to embracing the importance of these conversations and developing the knowledge, skills, and confidence to approach them with best practices. Key mechanisms through which staff report gaining confidence and skills to approach discussions include: (i) Alcohol ABC Approach training (including initial training sessions and refresher sessions which supported staff to come back and reflect on their initial conversations for further improvement and assurance); (ii) mentoring and support from Alcohol ABC Approach champions and colleagues; and (iii) having experience and familiarity with approaching conversations which enabled staff to feel less scripted and more naturalistic in their approach and delivery.

## Discussion

This process evaluation explored consumer and staff participant experiences and perceptions of having conversations about alcohol. Qualitative analyses generated five key themes: 1) “It’s a normal thing” - Social norms shape behaviour change and conversations about alcohol; 2) “It’s not a subject any stranger asks” - Whanaungatanga (relationships) and manaakitanga (hospitality) facilitate mana-enhancing conversations; 3) “It’s not just youth that are binge drinking” - Recognising implicit bias in conversations about alcohol; 4) “I need my health” - Conversations about alcohol are valuable and important, and; 5) “We are learning it is okay to ask”- Staff confidence to approach conversations about alcohol.

Alcohol use is inherently social; norms, expectations, behaviours, and beliefs surrounding alcohol are shaped by environmental, commercial, political, and socio-economic drivers that form the pervasive drinking culture across Aotearoa [24]. These drivers inform social norms and stigma around alcohol use, influencing both consumer and staff perceptions of risk, responsibility, and readiness to change alcohol use behaviours. Existing evidence suggests that personal readiness to change can be improved among patients with harmful alcohol use [25]. However, such research often fails to distinguish between individuals whose alcohol use may be perceived as socially acceptable and those who perceive their use as socially unacceptable, regardless of the level of harm. Consistent with previous studies, we found conversations about alcohol use may be particularly welcomed by consumers who identify as ‘ready’ to change [25–27], and consumer participants with more hazardous use may be more likely to report positive consumption changes [28]. Evaluation findings highlight the potential value of the Alcohol ABC Approach in supporting the de-normalisation of hazardous alcohol use, potentially priming readiness for future conversations [25].

While SBIRT often frames readiness to change alcohol as an individual psychological state [29], our evaluation highlights the limitations of this approach. Dominant Eurocentric models position change as a matter of personal responsibility and motivation [30], which may inadvertently reinforce self-blame and obscure social and structural influences on alcohol use behaviour. These models may not resonate with Māori, Pacific or other Indigenous peoples, for whom readiness may be relational and collectivist. A decolonised lens draws attention to these dynamics, recognising that readiness emerges relationally and contextually, shaped by family, community, and broader social, political, and economic environments. Within health systems, this lens shifts attention away from individualised deficit framings toward the role of service design and delivery, workforce practice, and institutional power in shaping how readiness is recognised and responded to [31]. Interrogating Eurocentric assumptions enables healthcare and public health approaches to critically re-evaluate how readiness and behaviour change around alcohol use may be understood and operationalised.

Decolonised approaches to alcohol harm reduction seek to shift power towards consumers and communities, aligning with Tiriti-based praxis that foregrounds power sharing, relational accountability, and anti-racism in health research and practice [32]. In the Aotearoa health context, decolonisation is increasingly understood as a system-wide transformation, requiring changes to how services engage Māori and their whānau, how care is delivered, and how equity obligations are enacted in everyday practice (reflecting calls for anti-racism, planned systems approaches to end institutional racism and uphold Te Tiriti obligations) [33]. By integrating relational and collectivist perspectives and valuing cultural practices such as whanaungatanga and manaakitanga, healthcare encounters can progress towards mana-enhancing and culturally safe interactions. Existing literature highlights the value and cultural imperative of such practices within healthcare to achieve equity [34–36]. Critically, they are central to the effective delivery of te Tiriti o Waitangi commitments by Crown agencies, including culturally safe and responsive public health services [37–39].

Staff practices can either mitigate or reproduce structural inequities. While many engage with positive intent, reliance on patient-initiated disclosure and unexamined implicit biases can limit access to care and reinforce power imbalances [40–49]. Within this evaluation, we identified missed opportunities for healthcare staff to connect with Māori in ways that more profoundly recognise Te Ao Māori and the position of Māori as Tangata whenua (indigenous people) in Aotearoa. Consistent with previous literature, implementation of tikanga Māori varied across clinical settings, with time-constrained and dynamic environments such as emergency departments presenting particular challenges for consumer-centred SBIRT [50,51]. Policy and system guidance in Aotearoa highlights the importance of workforce capability, cultural safety, and anti-racism competencies as foundational to decolonised health practice [52]. Embedding decolonial thinking, including critical reflection on positionality, cultural supervision, and structural awareness, offers a pathway to disrupt these inequities. Effective application of Tiriti-based praxis [31], incorporating relationally and culturally grounded practices, is critical to fulfilling equity obligations and enhancing consumer experiences.

Implicit biases, particularly ethnic bias (racism), remains a significant barrier. Evidence shows that misperceptions (e.g., judgement) and lack of connection between healthcare staff and consumers from minoritised ethnic groups contribute to negative outcomes [36,40,41]. Ethnic bias, entrenched through the historical and ongoing impacts of colonisation, influences clinical decision-making, policy development, and service delivery [42–45], shaping inequitable access, experience and outcomes for Māori and other underserved populations, as documented in national evidence syntheses [53]. Racism is a key determinant of health and has been identified as entrenched within health services in Aotearoa, with calls for systemic anti-racism action to address institutional inequities and uphold te Tiriti o Waitangi commitments [54,55]. Both over- and under-representation of ethnic groups within alcohol screening programmes demonstrates the potential for inequitable care [46,47]. This inequity can arise, in part, from staff avoiding conversations about alcohol, even when clinically indicated. Consistent with prior research, staff participants in this evaluation reported concerns that raising alcohol use could cause patient discomfort and negatively impact the therapeutic relationship [48–50]. Such concerns may be amplified when engaging with Māori and other underserved groups, underlining the importance of culturally safe, relationally grounded, and anti‑racist approaches in alcohol screening. Addressing racism requires staff to critically examine their own culture and positionality, recognise the subconscious influence of their cultural lens, and engage in structured interventions to identify and disrupt biased behaviours [56,57]. These measures are integral to operationalising decolonised approaches to alcohol harm reduction and ensuring equitable access to care.

Our findings highlight that decolonised approaches are essential for equitable delivery of the Alcohol ABC approach and alcohol harm reduction. Attending to relational, collective, and structural dimensions of readiness, and interrogating power and the impact of implicit bias within clinical encounters, enhances equity, supports meaningful trust building, and may mitigate harms associated with internalised self-blame. This has implications not only for clinical practice but also for public health responses, highlighting the importance of culturally safe, community-informed interventions.

### Evaluation strengths and limitations

The inclusion and prioritisation of consumer participant experiences and perspectives is a key strength of this evaluation. Insights from consumers are critical to better understanding how services can be improved. The key limitation pertaining to the qualitative aspects of the evaluation is the impact of COVID-19 on recruitment, contributing to only eight out of the intended 20 consumer interviews completed, and only by phone. Consequently, interviews were not whānau-inclusive as intended. These factors may have impacted on the diversity of experiences and perceptions included. Future evaluation should ensure a focused recruitment of low and moderate level alcohol use (i.e., AUDIT-C scores), as well as consumers across all health settings with strong participation by Māori and Pacific peoples and their whānau as priority populations.

## Conclusion

This evaluation highlights the critical importance of decolonised approaches to the Alcohol ABC Approach and alcohol harm reduction. Decolonising health and care systems in Aotearoa New Zealand are clearly aligned with health equity rights, needs, and aspirations of Māori communities, te Tiriti o Waitangi, and strategic priorities of the health system [52], with relevance for other countries seeking equitable and culturally safe approaches for screening of alcohol harms. These findings implore programmatic policies, systems, and processes to address implicit and explicit biases, particularly racism, among staff who deliver the Alcohol ABC Approach.

## Supporting information

S1 Checklist PLOS inclusivity in global research statement. (DOCX)

S1 Text Historical context summary. Critical historical context relating to Māori health inequities, including the impacts of colonisation and Te Tiriti o Waitangi, to support interpretation of the findings and public health implications.
(DOCX)

S2 Text Glossary of Te Reo Māori terms. Te reo Māori glossary providing English translations of Māori terms used throughout the manuscript.
(DOCX)
