# Supplementary material for: Having conversations about alcohol in frontline healthcare services: Experiences and perceptions of consumers and healthcare staff
Source: PLOS Glob Public Health. 2026 Aug 19;6(8):e0006893. doi: 10.1371/journal.pgph.0006893 (PMC13489459; doi:10.1371/journal.pgph.0006893)
Supplement: Supplementary file 2 [file pgph.0006893.s002.docx]

## **Historical context of alcohol harm inequities in Aotearoa**

He Whakaputanga o te Rangatiratanga o Nu Tireni, the Declaration of Independence (1835), is Aotearoa's first constitutional document, affirming tino rangatiratanga among Māori (Māori sovereignty or full and complete authority by Māori, of their lives, culture, land, and all treasured things - including health) in Aotearoa. This was followed by Te Tiriti o Waitangi in 1840, a significant agreement between the British Crown and Rangatira Māori (Māori chiefs) involving a proclamation of British governance in Aotearoa [Article 1 - Kāwanatanga], the reaffirmation of Māori ‘tino rangatiratanga’ [Article 2 – Tino Rangatiratanga] and a guarantee that Māori would have the queen’s protection and all rights and benefits accorded to British subjects. Importantly, Article One places obligations on the Crown to ensure fair and just governance of all people in Aotearoa. If this were upheld, the right for Māori to experience good and equitable health would be observed [8]. Further, Māori would enjoy the full and complete expression of tino rangatiratanga which, among other indicators, may be illustrated by Māori having free and clear decision making and choices about how they live and flourish as Māori within Aotearoa society.

After signing, the British Crown and settler governments have consistently failed to honour Te Tiriti o Waitangi illustrated by ongoing and devastating impacts on Māori communities. In the early 19th century, Māori made up nearly 100% of the population and had authority over all the land in Aotearoa. By the end of the century, due to land dispossession and marginalisation, Māori were described in Pākehā (European) newspapers as “a dying race.” These historical breaches have profoundly affected Māori health. The loss of land and failure by successive governments to recognise Māori rights to tino rangatiratanga has disrupted traditional ways of living, causing economic hardship, social dislocation, and cultural loss among Māori. Consequently, Māori have faced significant health inequities, including higher rates of chronic diseases, mental health issues, and lower life expectancy compared to non-Māori.

Over generations, Māori have called on the government to honour Te Tiriti o Waitangi commitments, actively protesting to reclaim their rights, land, and sovereignty. The health inequities seen today are directly linked to these historical and contemporary injustices and the ongoing struggle for Māori to regain their guaranteed rights and wellbeing. These rights of Māori in Aotearoa form the critical context for the design, delivery and evaluation of interventions to address alcohol harm.
