# Supplementary material for: Having conversations about alcohol in frontline healthcare services: Experiences and perceptions of consumers and healthcare staff
Source: PLOS Glob Public Health. 2026 Aug 19;6(8):e0006893. doi: 10.1371/journal.pgph.0006893 (PMC13489459; doi:10.1371/journal.pgph.0006893)
Supplement: Supplementary file 3 [file pgph.0006893.s003.docx]

**Glossary of Te Reo Māori terms**

This glossary is informed by article authors and the Te Aka Māori Dictionary (online). Terms are presented in alphabetical order.

*Aotearoa* – North Island - now used as the te reo Māori name for New Zealand.

*He Whakaputanga o te Rangatiratanga o Nu Tireni* - The Declaration of Independence, signed in 1835, declaring Māori national identity and sovereignty.

*Koha* – A gift or token of appreciation.

*Kōrero* – Conversation or discussion.

*Manaakitanga* – Care, respect, hospitality or kindness to others.

*Mana* – As detailed in the article, mana is a fundamental concept in Māori culture, representing authority, inherent power, dignity and spiritual strength.

*Mana*-*enhancing* – Refers to actions, practices, and policies that aim to uplift, empower, and respect the dignity, authority, and identity of individuals or groups.

*Māori* – An indigenous person of Aotearoa New Zealand, a term introduced through colonisation to distinguish between colonisers and indigenous peoples.

*Pākehā* – New Zealander of European descent.

*Rangatira* *Māori* – Māori chiefs.

*Tangata tiriti –* A term used to describe non-Māori people in Aotearoa, or people of the Treaty.

*Tangata whenua –* indigenous people.

*Te* *Ao* *Māori* – Māori worldviews or perspectives.

*Te* *Reo* *Māori* – The Māori language.

*Te* *Tiriti* *o* *Waitangi* – The Treaty of Waitangi - a significant agreement between the British Crown and Rangatira Māori (Māori chiefs) made in 1840.

*Tikanga* – Māori customs.

*Tino* *rangatiratanga* – Self-determination, sovereignty, autonomy.

*Whakawhanaungatanga* – The process of building culturally meaningful connections.

*Whānau* – Extended family.

*Whanaungatanga* – Relationship building.
